# The Scar of Suicide: Lasting Functional and Cognitive Impairment in Euthymic Bipolar type I Patients

**DOI:** 10.1192/j.eurpsy.2026.12081

**Published:** 2026-07-31

**Authors:** E. M. Bahri, S. Ellini, F. Ghrissi, W. Cherif, R. Damak, M. E. Cheour, F. F. Romdhane

**Affiliations:** 1 Espoir Addiction Service, Jbel Ouest Healthcare Complex, Zaghouan; 2 Psychiatry Department E, Razi Hospital, Manouba, Tunisia

## Abstract

**Introduction:**

Functional recovery remains a major unmet need in bipolar disorder (BD). While many patients achieve mood stability, a significant proportion continue to struggle with daily life. A history of suicide attempts is a key indicator of illness severity, but its specific relationship to functional and cognitive outcomes in euthymia is poorly understood.

**Objectives:**

We aimed to investigate the suicide attempt history relationship, and determine its use in risk identification.

**Methods:**

A case-control study was conducted involving 100 euthymic patients with BD Type I and 100 healthy controls. Participants were assessed using the Functioning Assessment Short Test (FAST) to measure functional impairment and the Cognitive Complaints in Bipolar Disorder Rating Assessment (COBRA) scale to measure subjective cognitive complaints. A history of suicide attempts was recorded through clinical interview. Statistical analyses included Mann-Whitney U tests to compare scale scores between patients with and without a suicide attempt history, and Spearman’s correlation for the number of attempts. A p-value <0.05 was considered significant. Data was collected and analyzed using SPSS 27 software.

**Results:**

The cohort of 100 euthymic BD type 1 patients was divided into two groups: those with a history of suicide attempts (SA+, n=50) and those without (SA-, n=50). The groups were well-matched on key sociodemographic and clinical variables, reinforcing that the outcome differences are likely attributable to the suicide history itself.

The groups were similar in age (SA+: 41.8±11.2 vs. SA-: 43.2±10.4 years) and gender, with a slight female majority (SA+: 28 women, 56% vs. SA-: 25 women, 50%). The proportion of unemployed patients was higher in the SA+ group (n=33, 66% vs. SA-: n=27, 54%). Educational attainment was balanced across levels. The median illness duration was long and comparable between groups (SA+: 19 years vs. SA-: 17 years). A high prevalence of psychotic symptoms was observed in both groups (SA+: n=44, 88% vs. SA-: n=41, 82%), as was a low rate of rapid cycling (SA+: n=6, 12% vs. SA-: n=4, 8%). None of these differences were statistically significant.

A history of suicide attempts was associated with significantly greater global functional impairment (Mean FAST score: 33.4 vs. 23.8; p=0.001). The same group also reported significantly higher levels of subjective cognitive complaints (Median COBRA score: 10 vs. 6; p=0.049). However, the number of suicide attempts did not correlate with scores on either scale (ρ=-0.082; p=0.572), indicating that the history itself is the critical factor, not frequency.

**Conclusions:**

In euthymic BD type 1, a history of suicide attempts—regardless of frequency—is a potent marker for severe functional disability and subjective cognitive deficits. This underscores an urgent need for integrated therapies targeting functional and cognitive recovery in this patient subgroup.

**Disclosure of Interest:**

None Declared

